# Utility of a Large Series of B‐Cell Precursor Acute Lymphoblastic Leukemia Cell Lines as a Model System

**DOI:** 10.1002/cam4.70736

**Published:** 2025-03-01

**Authors:** Minori Tamai, Chiaki Komatsu, Keiko Kagami, Shin Kasai, Koshi Akahane, Kumiko Goi, Kanji Sugita, Chihiro Tomoyasu, Toshihiko Imamura, Hiroaki Goto, Takeshi Inukai

**Affiliations:** ^1^ Global Leukemia Cell‐Line Assembly Network University of Yamanashi Yamanashi Japan; ^2^ Department of Pediatrics University of Yamanashi Yamanashi Japan; ^3^ Department of Pediatrics Graduate School of Medical Science, Kyoto Prefectural University of Medicine Kyoto Japan; ^4^ Hematology/Oncology Kanagawa Children's Medical Center Kanagawa Japan

**Keywords:** BCP‐ALL, cell cycle, chemoresistance, leukemogenesis, relapse, RNA‐seq

## Abstract

**Background:**

In B‐cell precursor acute lymphoblastic leukemia (BCP‐ALL), chromosomal translocations are strongly associated with prognoses. RNA sequencing (RNA‐seq) is a powerful technology that reveals a close correlation between types of translocation and patterns of gene expression in clinical samples of BCP‐ALL. Cancer cell lines are powerful research tools, and thus, we built a larger series of BCP‐ALL cell lines and performed RNA‐seq analysis to confirm their utility as a model system.

**Methods:**

We performed RNA‐seq in a total of 94 BCP‐ALL cell lines, including 80 cell lines with 8 representative types of translocations.

**Results:**

In the UMAP visualization, a close association was confirmed between the types of fusion genes and patterns of gene expression. In the cluster analysis of the gene expression profile, each type of fusion gene showed a clear association with the expression profile in the top 51 variable genes. Of clinical importance, the majority of the top variable genes in the BCP‐ALL cell lines also showed a significant association with the types of fusion genes in the clinical samples. When an association of 125 cell cycle‐related genes with the percentage of S and G2/M phases in 67 cell lines was evaluated, a significant positive correlation with cell cycle progression was confirmed in 10 cell cycle‐related genes (*HDAC2*, *CDC23*, *YWHAG*, *MAD2L1*, *CCNH*, *ANAPC7*, *CDC6*, *ANAPC5*, *ORC3*, and*RBX1*). Moreover, significant upregulation and downregulation of 40 and 10 genes, respectively, were observed in the cell lines established at relapse compared with those established at diagnosis. Four (*SP6*, *CCNE1*, *HIST1H2BH*, and *DECR2*) and two (*EVI2B* and *SYN1*) of these genes were also significantly higher and lower, respectively, in the clinical samples at relapse than in those at diagnosis.

**Conclusion:**

Large series of BCP‐ALL cell lines is a powerful research tool for studying the mechanisms of leukemogenesis and the disease progression of BCP‐ALL.

## Graphical abstract

RNA sequencing in a large series of B‐cell precursor acute lymphoblastic leukemia cell lines was performed. Using the data, a close association between types of fusion genes and patterns of gene expression was demonstrated. Furthermore, several cell cycle‐related genes that were correlated with cell cycle progression and relapsed‐related genes that were associated with disease relapse in the clinical samples were identified.

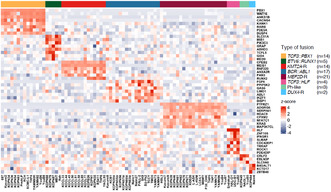

## 1 Introduction

In acute lymphoblastic leukemia (ALL), particularly in B‐cell precursor ALL (BCP‐ALL), chromosomal translocations, which are the most critical cytogenetic abnormalities, are highly associated with prognoses. Based on recent advances in next‐generation sequencing technologies, many studies using clinical samples have demonstrated the utility of RNA sequencing (RNA‐seq) in the molecular taxonomy of ALL [1, 2, 3, 4]. RNA‐seq is a powerful technology that enables us to simultaneously evaluate gene rearrangements and gene expression profiles in a single assay. As a result, RNA‐seq reveals a close correlation between types of gene rearrangement and patterns of gene expression in BCP‐ALL [1, 2, 3, 4].

Cancer cell lines are powerful research tools for a better understanding of the biology and therapeutic responses in cancers. In particular, a large series of cancer cell lines is an outstanding platform for studying genomic diversity and identifying novel therapeutic targets in human cancers. The Cancer Cell Line Encyclopedia (CCLE) systematically profiles more than 1000 cell lines from diverse lineages, and the Dependency Map (DepMap) portal derived from CCLE is the most widely used genetic database for many cancers [5, 6].

As indicated in the CCLE studies [5, 6], leukemic cell lines are also useful research tools. However, the BCP‐ALL cell lines available on the CCLE platform are currently limited to 36 cell lines. Despite the diverse genomic information for each cell line, this number might not be sufficient to evaluate a possible correlation between types of gene rearrangement and patterns of gene expression. Thus, we sought to build a larger series of BCP‐ALL cell lines as a model system to understand the mechanisms of leukemogenesis and drug resistance. Since most BCP‐ALL cell lines harbor chromosomal translocations, their gene expression profiles could show a significant association with their types of fusion genes. Thus, in the present study, we performed RNA‐seq analysis in our large series of BCP‐ALL cell lines and confirmed a close correlation between types of fusion genes and gene expression profiles. We further evaluated the association of the gene expression profile with cell proliferation and disease progression.

## 2 Materials and Methods

### 2.1 Cell Lines

We used 94 BCP‐ALL cell lines (Table 1), including 21 *MEF2D*‐rearranged (*MEF2D*‐R), 17 *BCR::ABL1*, 14 *KMT2A*‐R, 14 *TCF3::PBX1*, 5 *ETV6::RUNX1*, 4 *TCF3::HLF*, 3 Ph‐like, and 2 *DUX4*‐R cell lines. All cell lines were maintained in RPMI1640 medium supplemented with 10% fetal calf serum (FCS) in a humidified atmosphere of 5% CO_2_ at 37°C. Forty‐four cell lines had been sequentially established in our laboratory since 1978. In brief, heparinized sterile mononuclear cells isolated from peripheral blood or bone marrow by Ficoll density gradient centrifugation were cultured in RPMI1640 medium supplemented with 20% FCS until they proliferated exponentially. Research protocol, including written informed consent, was approved by the University of Yamanashi, Certified Review Board: Approval No. 1231.

**TABLE 1:** List of cell line.

Cell lines	Fusion gene	Establishment	S‐G2/M (%)	Calalog no.	CCLE
697	*TCF3::PBX1*	At relapse	NA	DSMZ ACC 42	○
Kasumi2^e^	*TCF3::PBX1*	At relapse	38	JCRB1395	○
KOPN34^a^	*TCF3::PBX1*	At diagnosis	43	NA	NA
KOPN36^a^	*TCF3::PBX1*	At relapse	49	NA	NA
KOPN54^a^	*TCF3::PBX1*	At relapse	32	NA	NA
KOPN63^a^	*TCF3::PBX1*	At relapse	50	NA	NA
PreALP	*TCF3::PBX1*	At diagnosis	NA	NA	NA
RCH	*TCF3::PBX1*	At relapse	NA	DSMZ ACC 548	NA
SCMC‐L1^l^	*TCF3::PBX1*	NA	37	NA	NA
THP4^c^	*TCF3::PBX1*	At diagnosis	28	NA	NA
YAMN90R^a^	*TCF3::PBX1*	At relapse	34	NA	NA
YAMN92^a^	*TCF3::PBX1*	At relapse	29	NA	NA
YcuB6^b^	*TCF3::PBX1*	At diagnosis	28	DSMZ ACC 966	NA
YcuB8^b^	*TCF3::PBX1*	At diagnosis	33	NA	NA
KOPN68^a^	*ETV6::RUNX1*	At relapse	30	NA	NA
KOPN79^a^	*ETV6::RUNX1*	At relapse	16	NA	NA
KOPN87^a^	*ETV6::RUNX1*	At relapse	32	NA	NA
MBIT^d^	*ETV6::RUNX1*	At diagnosis	NA	NA	NA
Reh	*ETV6::RUNX1*	At relapse	NA	ATCC CRL‐8286	○
KOCL33^a^	*KMT2A‐R (KMT2A::MLLT1)*	At diagnosis	51	NA	NA
KOCL44^a^	*KMT2A‐R (KMT2A::MLLT1)*	At relapse	34	NA	NA
KOCL45^a^	*KMT2A‐R (KMT2A::AFF1)*	At relapse	56	NA	NA
KOCL50^a^	*KMT2A‐R (KMT2A::AFF1)*	At relapse	57	NA	NA
KOCL51^a^	*KMT2A‐R (KMT2A::MLLT1)*	At diagnosis	35	NA	NA
KOCL58^a^	*KMT2A‐R (KMT2A::AFF1)*	At diagnosis	38	NA	NA
KOCL69^a^	*KMT2A‐R (KMT2A::AFF1)*	At relapse	43	NA	NA
KOCL77^a^	*KMT2A‐R (KMT2A::AFF1)*	At relapse	NA	NA	NA
KOPB26^a^	*KMT2A‐R (KMT2A::MLLT3)*	At relapse	57	NA	NA
KOPN1^a^	*KMT2A‐R (KMT2A::MLLT1)*	At relapse	41	NA	NA
KOPN35^a^	*KMT2A‐R (KMT2A::MLLT10)*	At relapse	48	NA	NA
RS411	*KMT2A‐R (KMT2A::AFF1)*	At relapse	NA	ATCC CRL‐1873	○
THP8^c^	*KMT2A‐R (KMT2A::AFF1)*	At diagnosis	32	NA	NA
YACL95	*KMT2A‐R (KMT2A::MLLT3)*	At diagnosis	40	NA	NA
Kasumi8^e^	*BCR::ABL1*	At relapse	NA	JCRB 1403	NA
KCB1^b^	*BCR::ABL1*	At diagnosis	11	NA	NA
KOPN30bi^a^	*BCR::ABL1*	At relapse	23	NA	NA
KOPN55bi^a^	*BCR::ABL1*	At relapse	15	NA	NA
KOPN56^a^	*BCR::ABL1*	At relapse	36	NA	NA
KOPN57bi^a^	*BCR::ABL1*	At diagnosis	34	NA	NA
KOPN66bi^a^	*BCR::ABL1*	At relapse	33	NA	NA
KOPN83bi^a^	*BCR::ABL1*	At relapse	24	NA	NA
Nalm1	*BCR::ABL1*	NA	NA	ATCC CRL‐1567	NA
Nalm27	*BCR::ABL1*	At diagnosis	37	JCRB 1830	NA
PALL‐2	*BCR::ABL1*	At relapse	NA	JCRB 1345	NA
SK9^j^	*BCR::ABL1*	At relapse	38	NA	NA
SU‐Ph2^g^	*BCR::ABL1*	At relapse	30	NA	NA
TCCY^h^	*BCR::ABL1*	NA	40	NA	NA
TMD5	*BCR::ABL1*	At diagnosis	NA	JCRB IFO50516	NA
YAMN73^a^	*BCR::ABL1*	At relapse	32	NA	NA
YAMN91^a^	*BCR::ABL1*	At diagnosis	34	NA	NA
HBL3^f^	*MEF2D*‐R (*MEF2D::BCL9*)	At relapse	NA	NA	NA
Kasumi7^e^	*MEF2D*‐R *(MEF2D::HNRNPUL1)*	At relapse	NA	JCRB 1401	NA
Kasumi9^e^	*MEF2D*‐R *(MEF2D::HNRNPUL1)*	At relapse	NA	JCRB 1409	NA
KOPN39^a^	*MEF2D*‐R (*MEF2D::BCL9*)	At diagnosis	44	NA	NA
KOPN46^a^	*MEF2D*‐R *(MEF2D::HNRNPUL1)*	At diagnosis	NA	NA	NA
KOPN61^a^	*MEF2D*‐R (*MEF2D::BCL9*)	At diagnosis	36	NA	NA
KOPN62^a^	*MEF2D*‐R (*MEF2D::BCL9*)	NA	38	NA	NA
KOPN70^a^	*MEF2D*‐R (*MEF2D::BCL9*)	At relapse	39	NA	NA
KOPN71^a^	*MEF2D*‐R (*MEF2D::BCL9*)	At diagnosis	42	NA	NA
KOS20	*MEF2D*‐R (*MEF2D::BCL9*)	At relapse	45	NA	NA
L‐ASK^c^	*MEF2D*‐R (*MEF2D::BCL9*)	At diagnosis	36	NA	NA
L‐KUM^c^	*MEF2D*‐R (*MEF2D::DAZAP1*)	At relapse	40	NA	NA
LC4‐1	*MEF2D*‐R (*MEF2D::HNRNPUL1*)	NA	NA	JCRB 0114	○
MBMY^d^	*MEF2D*‐R (*MEF2D::HNRNPUL1*)	At diagnosis	46	NA	NA
P30/OHK	*MEF2D*‐R (*MEF2D::HNRNPUL1*)	At relapse	43	RCB 1938	○
THP5^c^	*MEF2D*‐R (*MEF2D::HNRNPUL1*)	At diagnosis	44	NA	NA
THP7^c^	*MEF2D*‐R (*MEF2D::BCL9*)	At diagnosis	52	NA	NA
YAMN74^a^	*MEF2D*‐R (*MEF2D::BCL9*)	At relapse	41	NA	NA
YAMN96^a^	*MEF2D*‐R (*MEF2D::HNRNPUL1*)	At diagnosis	NA	NA	NA
YcuB4^b^	*MEF2D*‐R (*MEF2D::BCL9*)	At diagnosis	41	DSMZ ACC 962	NA
YcuB7^b^	*MEF2D*‐R (*MEF2D::BCL9*)	At relapse	33	NA	NA
Endokun^k^	*TCF3::HLF*	At relapse	39	NA	NA
HAL‐O1^i^	*TCF3::HLF*	At relapse	31	RCB 0540	○
UOC‐B1	*TCF3::HLF*	At relapse	NA	NA	NA
YcuB2^b^	*TCF3::HLF*	At relapse	46	DSMZ ACC 961	NA
KOPN49^a^	*IgH::CRLF2*	At relapse	24	NA	NA
NAGL‐1	*IgH::CRLF2*	NA	NA	JCRB IFO50479	NA
YcuB5^b^	*P2RY8::CRLF2*	At diagnosis	19	DSMZ ACC 964	NA
KOPN84^a^	*DUX4*‐R	At relapse	43	NA	NA
Nalm6	*DUX4*‐R	At relapse	NA	ATCC CRL‐3273	○
CCRF‐SB	Others	NA	NA	JCRB 0032	NA
KCB2^b^	Others	At relapse	NA	NA	NA
KCB4^b^	Others	At relapse	21	NA	NA
KCB6^b^	Others	NA	NA	NA	NA
KCB7^b^	Others	At diagnosis	NA	NA	NA
KOPB38^a^	Others	NA	NA	NA	NA
KOPB59^a^	Others	NA	NA	NA	NA
KOPN32^a^	Others	At relapse	41	NA	NA
KOPN40^a^	Others	At relapse	34	NA	NA
KOPN75^a^	Others	At diagnosis	45	NA	NA
KOPN85^a^	Others	At relapse	28	NA	NA
MBKG^d^	Others	At relapse	54	NA	NA
MBOK^d^	Others	At relapse	48	NA	NA
SCMC‐L2^l^	Others	NA	49	NA	NA

Abbreviations: ATCC, American Type Culture Collection; DSMZ, German Collection of Microorganisms and Cell Cultures; JCRB, Japanese Collection of Research Bioresources; NA, not abailable; RCB, RIKEN Cell Bank.

^a^ In house.

^b^ Yokohama City University and Kanagawa Children's Medical Center (Dr. H. Goto).

^c^ Tohoku University (Dr. M. Minegishi).

^d^ Mie University Graduate School of Medicine (Dr. S. Iwamoto).

^e^ Hiroshima University (Dr. T. Inaba).

^f^ Fukushima Medical University (Dr. H. Hojo).

^g^ Kindai University Faculty of Medicine (Dr. Y. Maeda).

^h^ Tochigi Cancer Center (Dr. Y. Sato).

^i^ Dana‐Farber Cancer Institute, Boston, MA (Dr. A. T. Look).

^j^ Tokyo Medical University (Dr. S. Okabe).

^k^ Iwate Medical University (Dr. M. Endo).

^l^ Saitama Children's Medical Center (Dr. J. Takita).

### 2.2 RNA‐Seq Analysis

RNA‐seq was performed by Rhelixa Co. Ltd. (Tokyo, Japan) using total RNA extracted with a RNeasy Plus Mini Kit (QIAGEN, Hilden, Germany). Libraries were prepared using a NEBNext Ultra II Directional RNA Library Prep Kit (New England BioLabs, Beverly, MA) and then sequenced on an Illumina NovaSeq 6000 with 150 × 2 paired‐end reads. Reads were trimmed to remove adapters and low‐quality bases with the TrimGalore! (v.0.6.6) graphical interface tool. The trimmed reads were aligned to the reference genome (hg38) using the RNA‐seq aligner STAR (v.2.1.0) with RSEM (v.1.1.17), according to a previous report [7]. The total number of reads and gene lengths among the samples were corrected using transcripts per million (TPM). Differentially expressed genes between the two groups were analyzed using the R package DEseq2 (v.1.38.3). For two‐dimensional UMAP, which was performed by using the R package umap (v.0.2.10.0), variable genes were determined by analyzing differentially expressed genes between the cell lines with and without each fusion gene. We carried out fusion gene detection using FusionCatcher (v1.33) with default parameters [8]. The analysis was performed on raw fastq file of each cell line, utilizing the human genome reference GRCh38, and a summary of matrix is presented in Table S1.

### 2.3 Cell Cycle Analysis

Each cell line was fixed with 70% ethanol 1 day after medium exchange, stained with propidium iodide (PI) (Sigma, St. Louis, MO), and analyzed by flow cytometry. The median percentages of the S and G2/M phases in three independent analyses were previously determined in 67 cell lines [9].

### 2.4 Statistical Analyses

Simple linear regression of gene expression and cell cycle was performed using GraphPad Prism (v.10.0.2) software. The Mann–Whitney *U* test was applied to compare the two groups.

## 3 Results

### 3.1 Association of Types of Fusion Gene With Gene Expression Profile in BCP‐ALL Cell Lines

**FIGURE 1:**
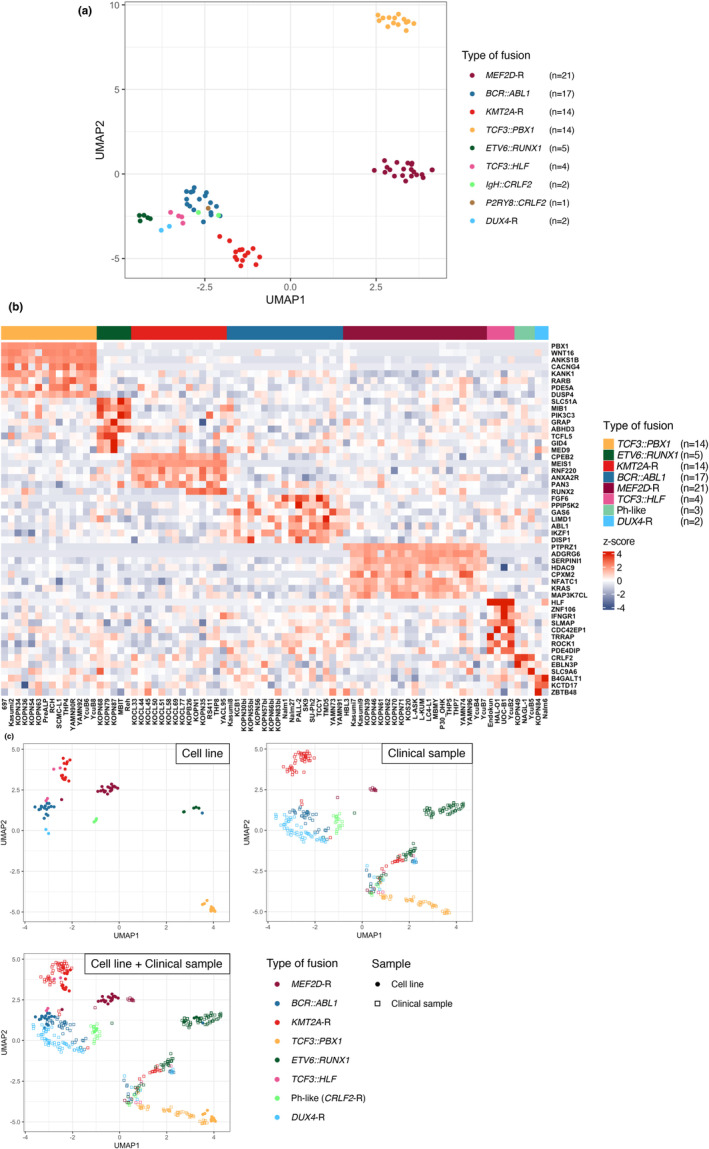
Association between types of fusion genes and patterns of gene expression in 80 BCP‐ALL cell lines with representative fusion genes. (a) Two‐dimensional UMAP visualization of gene expression profiles using the top 1554 variable genes in 80 BCP‐ALL cell lines with 8 representative types of fusion genes. Each dot indicates a single cell line, and the color of the dot indicates its type of fusion gene as follows: *MEF2D*‐R (dark red), *BCR::ABL1* (blue), *KMT2A*‐R (red), *TCF3::PBX1* (yellow), *ETV6::RUNX1* (moss green), *TCF3::HLF* (pink), *IgH::CRLF2* (light green), *P2RY8::CRLF2* (brown), and *DUX4*‐R (light blue). (b) Heat map of top 51 variable gene expressions in 80 BCP‐ALL cell lines with 8 representative fusion genes. Each column represents a single cell line. Types of fusion genes are indicated by colors on the top of the panel as follows: *TCF3::PBX1* (yellow), *ETV6::RUNX1* (moss green), *KMT2A*‐R (red), *BCR::ABL1* (blue), *MEF2D*‐R (dark red), *TCF3::HLF* (pink), Ph‐like (light green), and *DUX4*‐R (light blue). Red and blue color scaling indicates degrees of upregulation and downregulation of the mean expression across samples, respectively. (c) Two‐dimensional UMAP visualization of gene expression profile using the 48 variable genes in 80 BCP‐ALL cell lines and 341 childhood BCP‐ALL cases with 8 representative types of fusion genes. Each dot and square indicate a single cell line and a single case, respectively, and the color of each symbol indicates its type of fusion gene as the same as the above (a).

To confirm whether the types of fusion genes show an association with the gene expression profile in BCP‐ALL cell lines, we focused on 80 cell lines whose fusion genes were previously determined by RNA capture sequencing analysis [9]. These cell lines harbor the following eight representative types of fusion genes: *MEF2D*‐R, *BCR::ABL1*, *KMT2A*‐R, *TCF3::PBX1*, *ETV6::RUNX1*, *TCF3::HLF*, Ph‐like, and *DUX4*‐R. First, RNA‐seq identified the type of fusion gene in each cell line, which was the same as the one previously determined. Then, we performed UMAP analysis based on the 1554 most variable genes. Two‐dimensional UMAP (Figure 1a) visualized that *TCF3::PBX1* cell lines (yellow), *MEF2D*‐R cell lines (dark red), *KMT2A*‐R cell lines (red), and the other cell line types exclusively clustered with each other. Moreover, although relatively close to each other in visualization, *ETV6::RUNX1* cell lines (moss green), *DUX4*‐R cell lines (light blue), *TCF3::HLF* cell lines (pink), and *BCR::ABL1* cell lines (blue) showed exclusive expression profiles. Of note, the gene expression profile in the three Ph‐like cell lines [two *IgH::CRLF2* (light green) and one *P2RY8::CRLF2* (brown) cell lines] completely overlapped with those in the *BCR::ABL1* (blue) cell lines, as previously observed in the clinical samples [3, 4].

Next, we applied the cluster analysis to further investigate an association of the gene expression profile with 8 representative types of fusion genes in 80 cell lines. As shown in the heatmap based on the cluster analysis (Figure 1b), each type of fusion gene showed a clear association with the expression profile in the top 51 variable genes. The specific higher expression of *PBX1* in *TCF3::PBX1* cell lines, *ABL1* in *BCR::ABL1* cell lines, *HLF* in *TCF3::HLF* cell lines, and *CRLF2* in Ph‐like cell lines is assumed to be derived from reads of the fusion genes. To confirm the significance of these genes in the clinical samples, we developed a gene expression heatmap of the same genes in 341 childhood BCP‐ALL cases using the RNA‐seq data in a public database of St. Jude Children's Research Hospital (https://pecan.stjude.cloud/). We evaluated 48 genes except for *EBLN3P*, *HLF*, and *PAN3*, which were specifically associated with Ph‐like (*CRLF2*‐R), *TCF3::HLF*, and *KMT2A*‐R cell lines, respectively, due to lack of expression data. As shown in Figure S1, the majority of these genes showed a significant association with the types of fusion genes, particularly in *TCF3::PBX1*, *ETV6::RUNX1*, *KMT2A*‐R, and *MEF2D*‐R samples. Subsequently, we performed UMAP analysis of cell lines and clinical samples using these 48 variable genes (Figure 1c) and confirmed almost the same patterns of association between cell lines and clinical samples. These findings in our series of BCP‐ALL cell lines reveal a close association between the types of fusion genes and patterns of gene expression, consistent with the findings in the clinical samples [3, 4].

### 3.2 Association of Cell Cycle‐Related Genes With Cell Cycle Progression in BCP‐ALL Cell Lines

**FIGURE 2:**
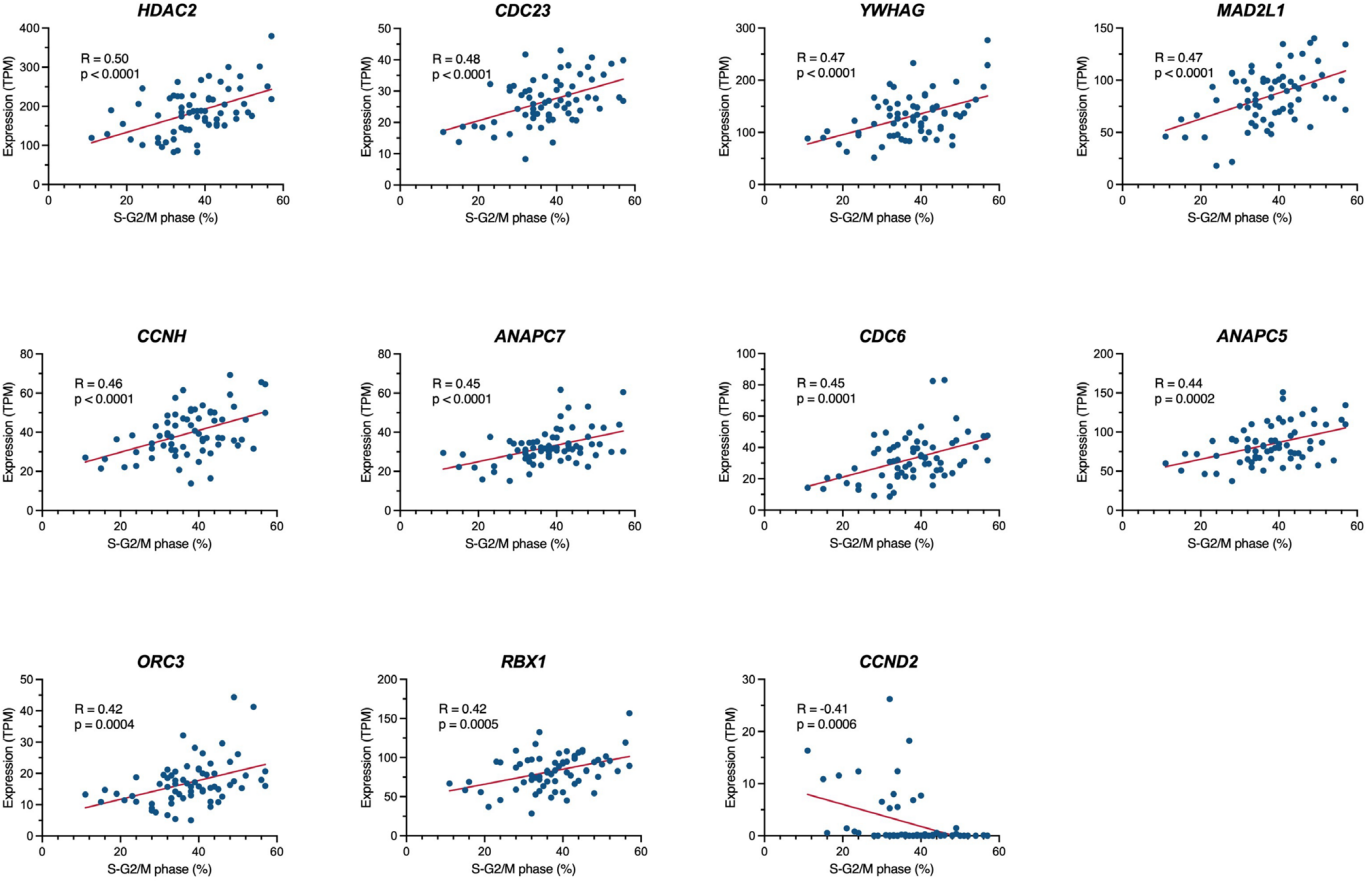
Association of cell cycle‐related gene expressions with cell cycle progression in BCP‐ALL cell lines. Each panel indicates the correlation of gene expression level (TPM value, vertical axis) to the percentage of the cells in the S and G2/M phases (horizontal axis) in 67 BCP‐ALL cell lines. The coefficient of correlation and p‐value are indicated in the upper left.

Leukemic cell lines generally show proliferative potential in vitro, which may at least partly be associated with the proliferative potential of leukemia cells in vivo. Thus, we applied the RNA‐seq data to identify the cell cycle‐related genes that are involved in the cell cycle progression of BCP‐ALL cell lines. We evaluated the association of 125 cell cycle‐related genes annotated in the KEGG pathway database (hsa04110) with the percentage of S and G2/M phases in 67 BCP‐ALL cell lines, in which cell cycle analysis was previously performed by PI staining. We found a significant positive correlation (*r* > 0.4) in 10 genes (Figure 2), including *HDAC2*, *CDC23*, *YWHAG*, *MAD2L1*, *CCNH*, *ANAPC7*, *CDC6*, *ANAPC5*, *ORC3*, and *RBX1*. In contrast, a significant negative correlation (*r* < −0.4) was unexpectedly observed in *CCND2*, which encodes cyclin D2 (Figure 2). These observations suggest that at least some of these genes might be involved in the cell cycle progression of BCP‐ALL cell lines.

### 3.3 Identification of Genes Associated With Disease Progression in BCP‐ALL

**FIGURE 3:**
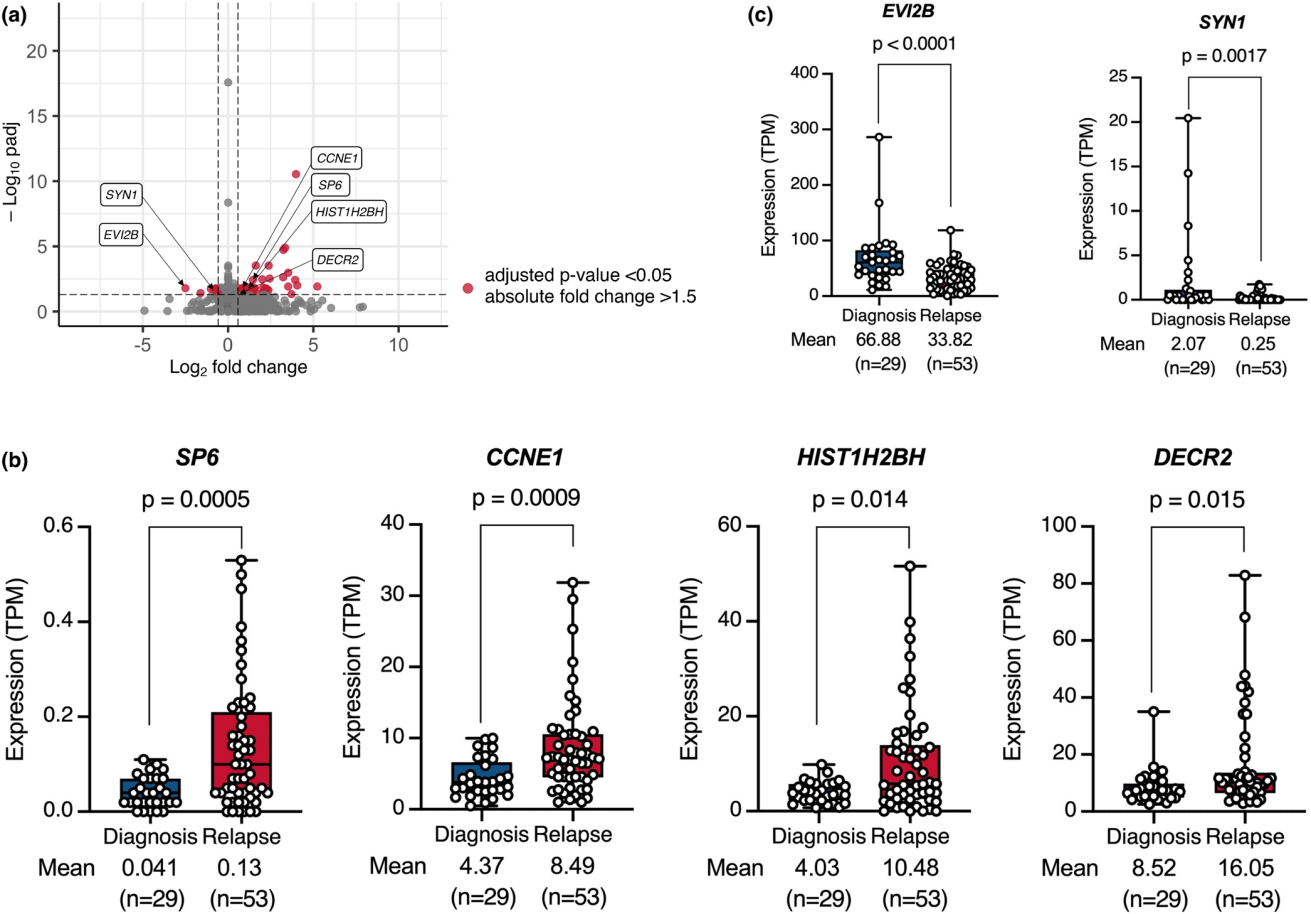
Relapse‐related gene expression in BCP‐ALL cell lines. (a) Volcano plot of differentially expressed genes between 29 cell lines established at diagnosis and 53 cell lines established at relapse. Each red plot indicates a single gene with an adjusted *p* < 0.05 and an absolute fold change > 1.5. (b and c) Comparison of the expression level of four upregulated (b) and two downregulated (c) genes between the cell lines established at diagnosis and those established at relapse.

Because the relapsed cases had poor prognoses, identification of the gene expression profile associated with relapse is important for improved therapeutic outcomes. Using 82 BCP‐ALL cell lines with establishment information, we compared gene expression profiles between cell lines established at diagnosis (*n* = 29) and those established at relapse (*n* = 53). As visualized in a volcano plot (Figure 3a), we identified 40 and 10 genes that were significantly upregulated and downregulated, respectively, in the cell lines established at relapse. Then, we compared the expression levels of these 50 genes between the clinical samples at diagnosis and those at relapse using a public database at St. Jude Children's Research Hospital. Of note, as observed in our BCP‐ALL cell lines (Figure 3b,c), four (*SP6*, *CCNE1*, *HIST1H2BH*, and *DECR2*) and two (*EVI2B* and *SYN1*) genes were significantly higher and lower in the samples at relapse than in those at diagnosis, respectively (Figure S2). Moreover, similar patterns of difference were separately observed in the cell lines with four representative types of fusion genes (Figure S3). Accordingly, at least some of these genes might be involved in disease progression.

## 4 Discussion

In the present study, RNA‐seq analysis was performed in a large number of BCP‐ALL cell lines. In the two‐dimensional UMAP analysis, 80 cell lines harboring 8 representative fusion genes were exclusively clustered with each other. In particular, we observed that the gene expression profiles of three Ph‐like cell lines, including two *IgH::CRLF2* and one *P2RY8::CRLF2* cell line, overlapped those of *BCR::ABL1* cell lines, as previously demonstrated in the clinical samples [3, 4]. Moreover, in the cluster analysis of the gene expression profile of 80 cell lines with 8 representative fusion genes, each type of fusion gene showed a clear association with the expression profile in the top 51 variable genes.

In the present study, by using the FusionCatcher, we failed to detect fusion genes in four cell lines, in which major types of fusion transcripts were already detectable by the RT‐PCR analysis. Thus, simultaneous application of other fusion detectors such as the STAR‐Fusion might be helpful to improve sensitivity. Among the 14 cell lines without 8 major types of fusion genes, no specific fusion genes were found in 11 cell lines, while the significance of the identified fusion gene in the remaining 3 cell lines is currently under investigation.

Of note, among these top variable genes, *WNT16* [10], *SLC51A* [11], *MEIS1* [12], and *PTPRZ1* [13] were reportedly specifically overexpressed in *TCF3::PBX1*, *ETV6::RUNX1*, *KMT2A*‐R, and *MEF2D*‐R clinical samples, respectively. Of clinical importance, the majority of these top variable genes in BCP‐ALL cell lines also showed a significant association with the types of fusion genes in the clinical samples when applied to the RNA‐seq data in a public database of 341 childhood BCP‐ALL cases at St. Jude Children's Research Hospital. To the best of our knowledge, although it has already been confirmed in clinical samples [3, 4], this is the first study confirming a close association between types of fusion genes and patterns of gene expression in a large series of BCP‐ALL cell lines. Additionally, we re‐investigated the association of fusion gene types with copy number alterations (Figure S4), which were previously evaluated by multiplex ligation‐dependent probe amplification analysis in the majority of cell lines [14]. As investigated in previous studies of clinical samples [15, 16], we confirmed the exclusive association of *IKZF1* deletion in *BCR::ABL1* cell lines and fewer copy number alterations in *KMT2A*‐R cell lines. These observations suggest similarities in the patterns of copy number alteration between BCP‐ALL cell lines and clinical samples. Accordingly, our observations indicate that BCP‐ALL cell lines are useful tools to investigate the biological significance of types of fusion genes, at least in terms of gene expression profiles.

We next applied the RNA‐seq data to identify the cell cycle‐related genes that are involved in the cell cycle progression of BCP‐ALL cell lines and identified 10 genes, including *HDAC2*, *CDC23*, *YWHAG*, *MAD2L1*, *CCNH*, *ANAPC7*, *CDC6*, *ANAPC5*, *ORC3*, and *RBX1*, which were positively correlated with cell cycle progression. Among these 10 genes, our literature search revealed the involvement of the following 6 genes in the cell cycle progression of cancers.

*HDAC2*, a histone deacetylase, reportedly promoted cell cycle progression of breast cancer cell lines, since CRISPR/Cas9‐mediated knockout induced reduction of the cells in S and G2/M phases [17]. *CDC23*, one of the APC subunits involving cell mitosis, was reportedly associated with cell cycle progression in liver cancer cell lines, since shRNA‐mediated knockdown repressed cell proliferation [18]. *YWHAG*, one of the 14‐3‐3 phospho‐serine/phospho‐threonine binding protein lines, promoted cell proliferation in the pancreas cancer cell lines, since expression vector‐mediated overexpression enhanced cell growth [19].

*MAD2L1*, a component of the mitotic spindle assembly checkpoint, was reportedly involved in cell cycle progression of colon cancer cell lines, since siRNA‐mediated knockdown induced a reduction of the cells in the S and G2/M phases [20]. *CDC6*, a DNA replication initiation factor, reportedly promoted cell cycle progression, since siRNA‐mediated knockdown blocked the G1/S transition in the HeLa cell line [21]. *RBX1*, an E3 ubiquitin ligase activator, reportedly promoted cell cycle progression in melanoma and multiple myeloma cell lines, since lentiviral vector‐mediated overexpression enhanced progression into the S phase [22].

Although no previous publications regarding involvement in tumorigenesis exist, the other four genes (*CCNH*, *ANAPC7*, *ANAPC5*, and *ORC3*) seem to be functionally consistent with cell cycle progression. On the other hand, we identified that the *CCND2* gene had a significant negative correlation with cell cycle progression in BCP‐ALL cell lines. However, 11 out of 15 cell lines with higher *CCDN2* expression (TPM value > 5) were *BCR::ABL1* cell lines, in which cyclin D2 is reportedly upregulated by the tyrosine kinase activity of the BCR::ABL1 protein [23, 24]. Thus, a negative correlation between cell cycle progression and *CCND2* might be simply due to a relatively slower cell cycle progression in *BCR::ABL1* cell lines. Taken together, at least some of these genes might be involved in the cell cycle progression of BCP‐ALL cell lines and, subsequently, BCP‐ALL cells in vivo, although further direct evaluation is required.

Finally, we compared gene expression profiles between cell lines established at diagnosis and those established at relapse and identified 40 and 10 genes that were significantly upregulated and downregulated, respectively, in the cell lines established at relapse. As a potential limitation of this analysis, clonal selection could occur during establishment in the cell lines established at diagnosis. Among those genes, we confirmed that 4 genes, including *SP6*, *CCNE1*, *HIST1H2BH*, and *DECR2*, and 2 genes, including *EVI2B* and *SYN1*, were significantly higher and lower in the clinical samples at relapse than in those at diagnosis, respectively. Of interest, our literature search revealed the possible involvement of all four upregulated and one of two downregulated genes in tumor progression. *SP6*, a Krüppel‐like family transcription factor, was reportedly involved in cell proliferation in dental pulp mesenchyme by enhancing canonical Wnt/β‐catenin signaling, since expression vector‐mediated overexpression induced cellular accumulation of β‐catenin [25]. *CCNE1*, a member of the cyclin family, was reportedly involved in cell cycle progression in breast cancer patients, since higher expression was associated with clinical resistance to a CDK4/6 inhibitor [26]. *HIST1H2BH*, a member of the H2B histone family, was reportedly involved in cell proliferation in multiple myeloma cell lines, since siRNA‐mediated knockdown inhibited proliferation [27]. *DECR2*, a 2,4‐dienoyl‐CoA reductase 2, was reportedly involved in cell proliferation in prostate cancer cell lines, since lentiviral vector‐mediated overexpression intensified cell growth [28].

Conversely, *EVI2B*, a single‐pass type I transmembrane glycoprotein, was reportedly involved in tumor immunity, since the cytotoxic activity of CD8+ T cells was intensified in melanoma patients with its higher expression [29]. Taken together, at least some of these genes might be involved in the disease progression of BCP‐ALL and, subsequently, poor outcomes in the relapsed cases, although further evaluation is required.

## 5 Conclusion

In the present study, using RNA‐seq analysis of large series of BCP‐ALL cell lines, we first demonstrated a close association between types of fusion genes and patterns of gene expression. We also identified a couple of cell cycle‐related genes that were positively correlated with cell cycle progression and relapsed‐related genes that were associated with disease relapse in the clinical samples. Although full multi‐omics analyses would be more informative, these observations revealed that our large series of BCP‐ALL cell lines is a powerful research tool for studying the mechanisms of leukemogenesis and the disease progression of BCP‐ALL.

## Author Contributions

**Minori Tamai:** conceptualization (lead), data curation (lead), formal analysis (lead), funding acquisition (lead), investigation (lead), methodology (lead), software (lead), validation (lead), visualization (lead), writing – original draft (lead). **Chiaki Komatsu:** formal analysis (supporting), investigation (supporting). **Keiko Kagami:** formal analysis (supporting), investigation (supporting). **Shin Kasai:** data curation (supporting). **Koshi Akahane:** data curation (supporting). **Kumiko Goi:** data curation (supporting). **Kanji Sugita:** data curation (supporting), resources (lead). **Chihiro Tomoyasu:** data curation (supporting), investigation (supporting), methodology (supporting). **Toshihiko Imamura:** data curation (supporting), investigation (supporting), methodology (supporting). **Hiroaki Goto:** data curation (supporting), resources (lead). **Takeshi Inukai:** conceptualization (supporting), data curation (supporting), funding acquisition (lead), investigation (supporting), methodology (supporting), project administration (lead), resources (lead), supervision (lead), validation (supporting), visualization (supporting), writing – review and editing (lead).

## Ethics Statement

Approval of the research protocol, including written informed consent, by the University of Yamanashi, Certified Review Board: Approval No. 1231 (2018 July 9th).

## Conflicts of Interest

The authors declare no conflicts of interest.

## Supporting information


Table S1.

Figure S1.

## Data Availability

The RNA‐seq data of 80 BCP‐ALL cell lines with 8 major fusion genes are available under the DDBJ BioProject database: PRJDB18849.
