# Supplementary material for: Utility of a Large Series of B‐Cell Precursor Acute Lymphoblastic Leukemia Cell Lines as a Model System
Source: Cancer Med. 2025 Mar 1;14(5):e70736. doi: 10.1002/cam4.70736 (PMC11871424; doi:10.1002/cam4.70736)

Fig. S1. Heat map of top 48 variable gene expression in 341 BCP-ALL clinical samples with 8 representative fusion genes. Using the PeCan database of St. Jude Children’s Research Hospital (https://pecan.stjude.cloud/), we evaluated the association between type of fusion gene and gene expression profile. Each column represents a single sample. Types of fusion gene are indicated by colors on the top of the panel as follows: *TCF3::PBX1* (yellow), *ETV6::RUNX1* (moss green), *KMT2A*-R (red), *BCR::ABL1* (blue), *MEF2D*-R (dark red), *TCF3::HLF* (pink), Ph-like with *CRLF2*-R (light green), and *DUX4*-R (light blue). Red and blue color scaling indicates degrees of upregulation and downregulation of the mean expression across samples, respectively.


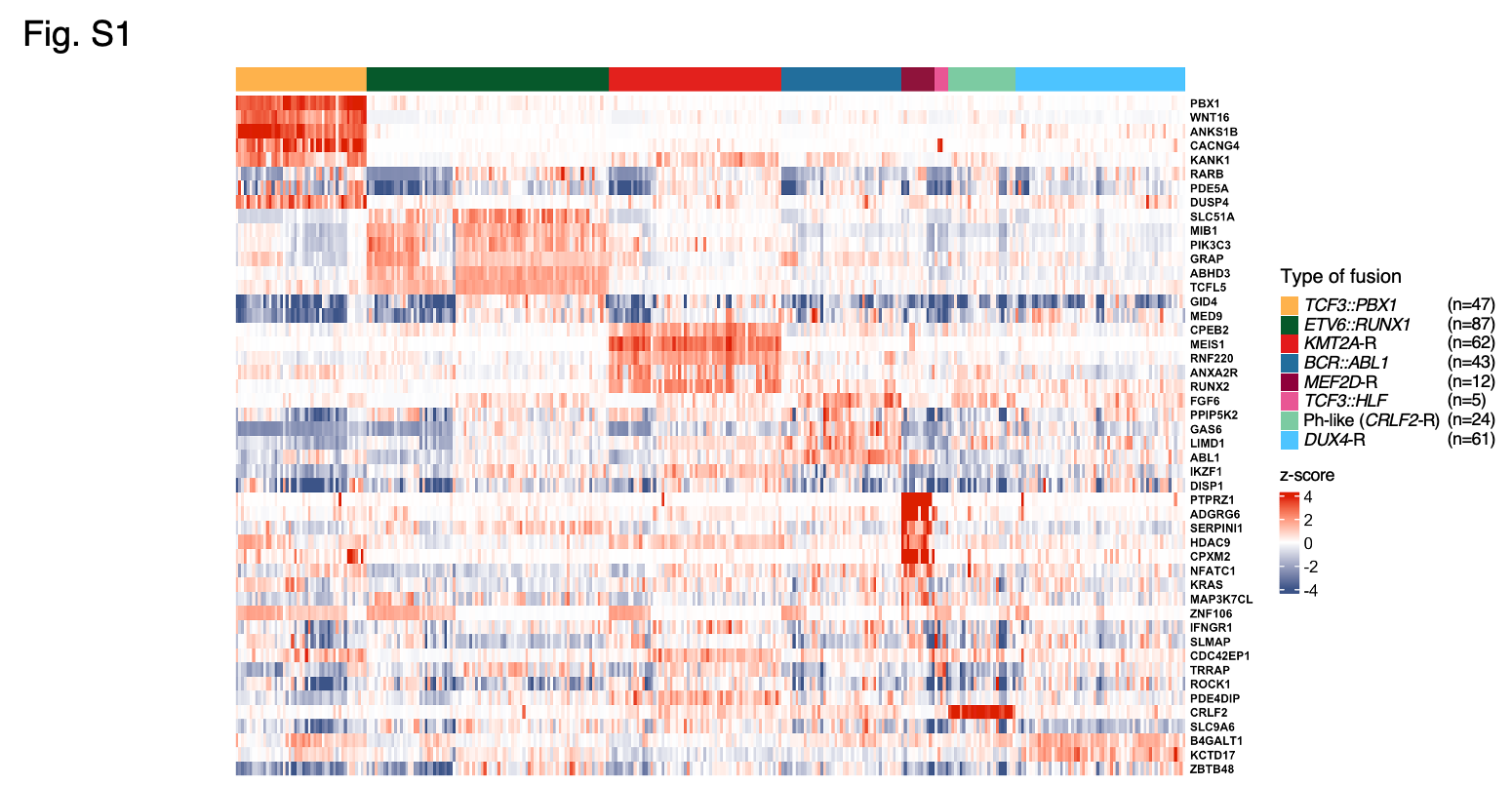


Fig. S2. Relapse-related gene expression in clinical samples. Comparison of expression level of four upregulated and two downregulated genes between the clinical samples at diagnosis and those at relapse. Using the PeCan database of St. Jude Children’s Research Hospital (https://pecan.stjude.cloud/), we evaluated the association between sample type (diagnosis or relapse) and gene expression profile.


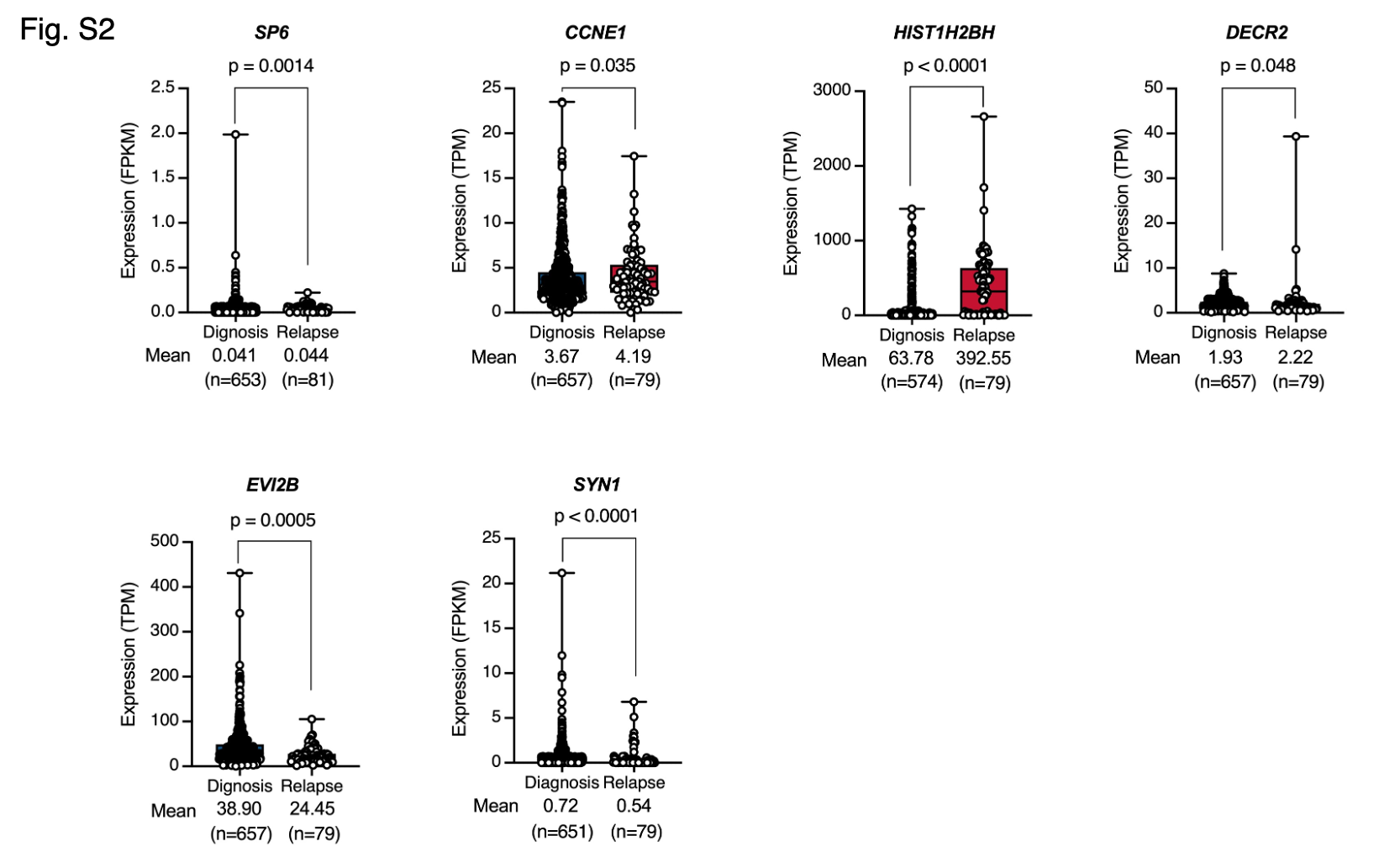


Fig. S3. Relapse-related gene expression in BCP-ALL cell lines with four representative types of fusion genes. (a-f) Comparison of expression level of four upregulated (a-d) and two downregulated (e, f) genes between the cell lines established at diagnosis and those established at relapse separately in the cell lines with four representative types of fusion genes (*KMT2A*-R, *BCR::ABL1*, *TCF3::PBX1*, and *MEF2D*-R).


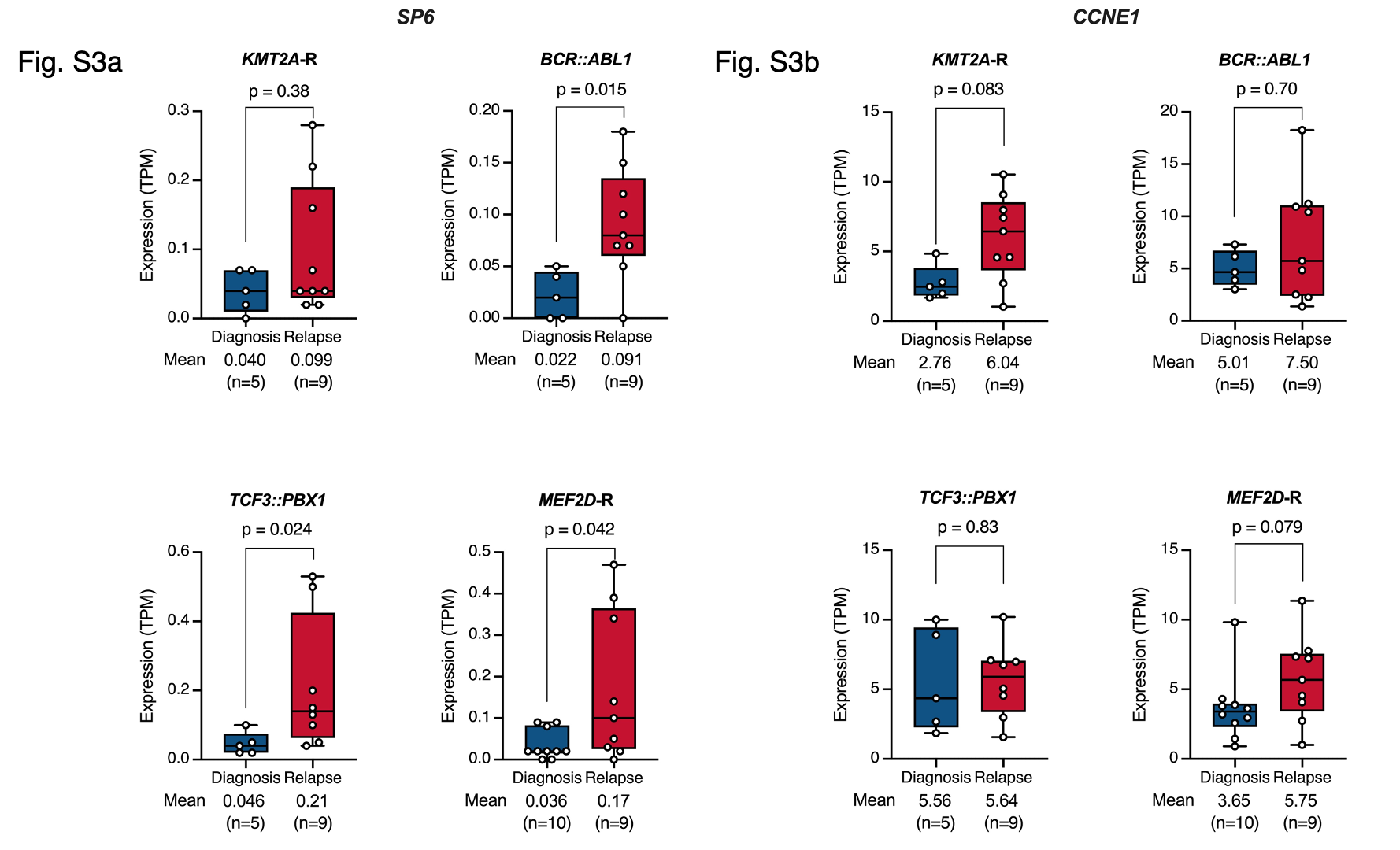


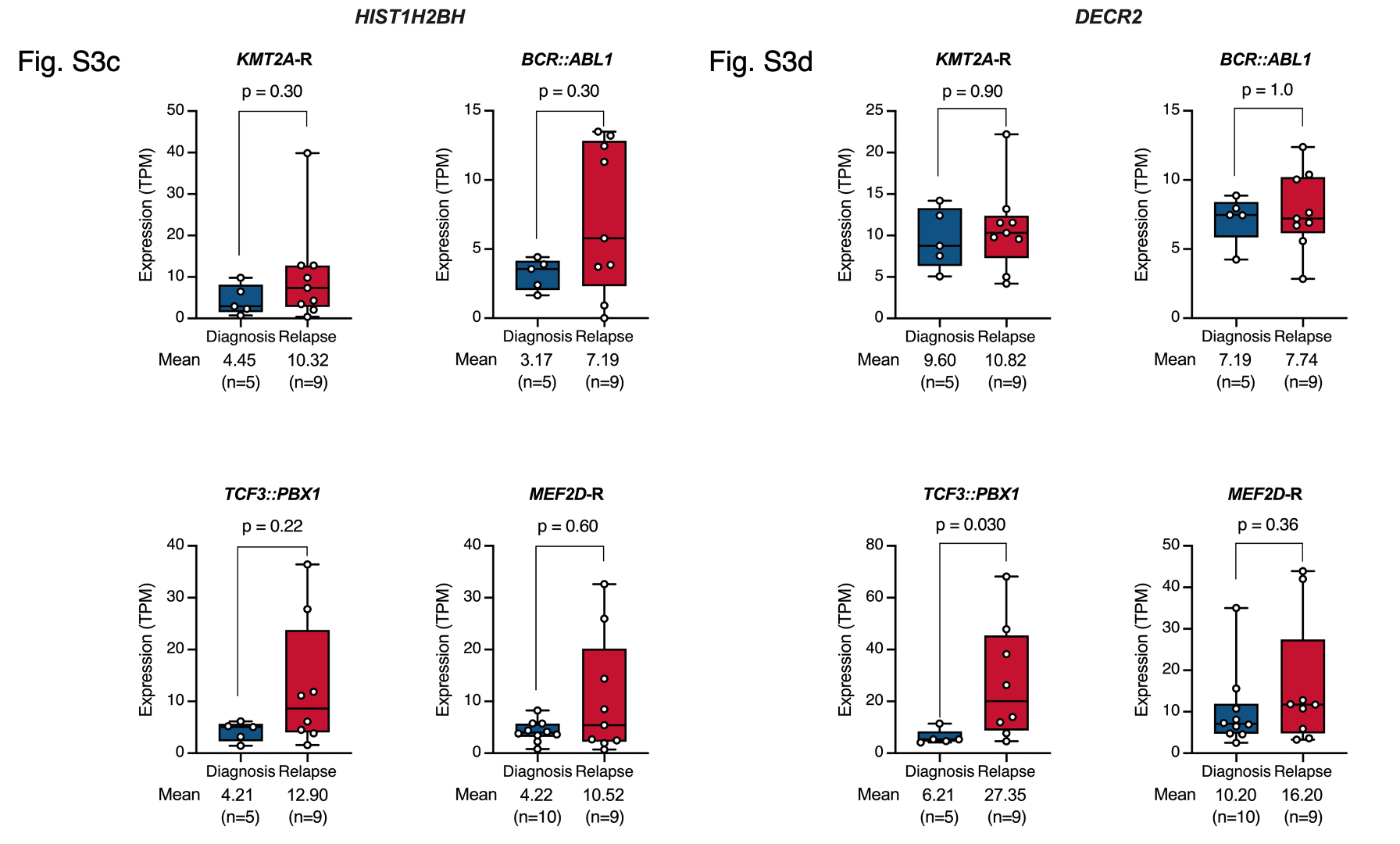


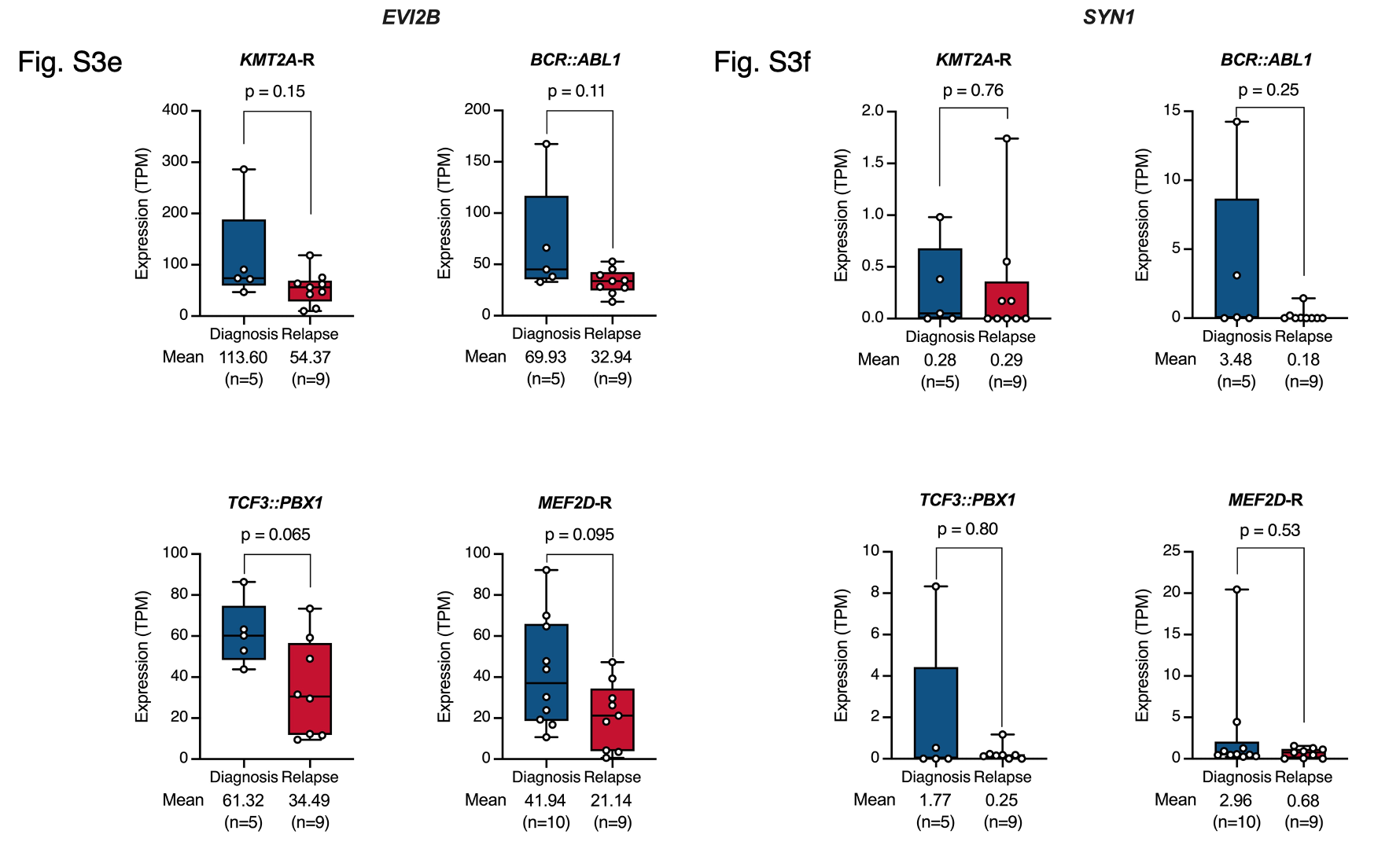


Fig. S4. Heatmap of 9 representative copy number alterations (rows) across 83 BCP-ALL cell lines (columns). In the panel, types of deletion (biallelic or monoallelic) are indicated by black and gray, respectively. In the top panel, types of fusion gene are indicated by colors.


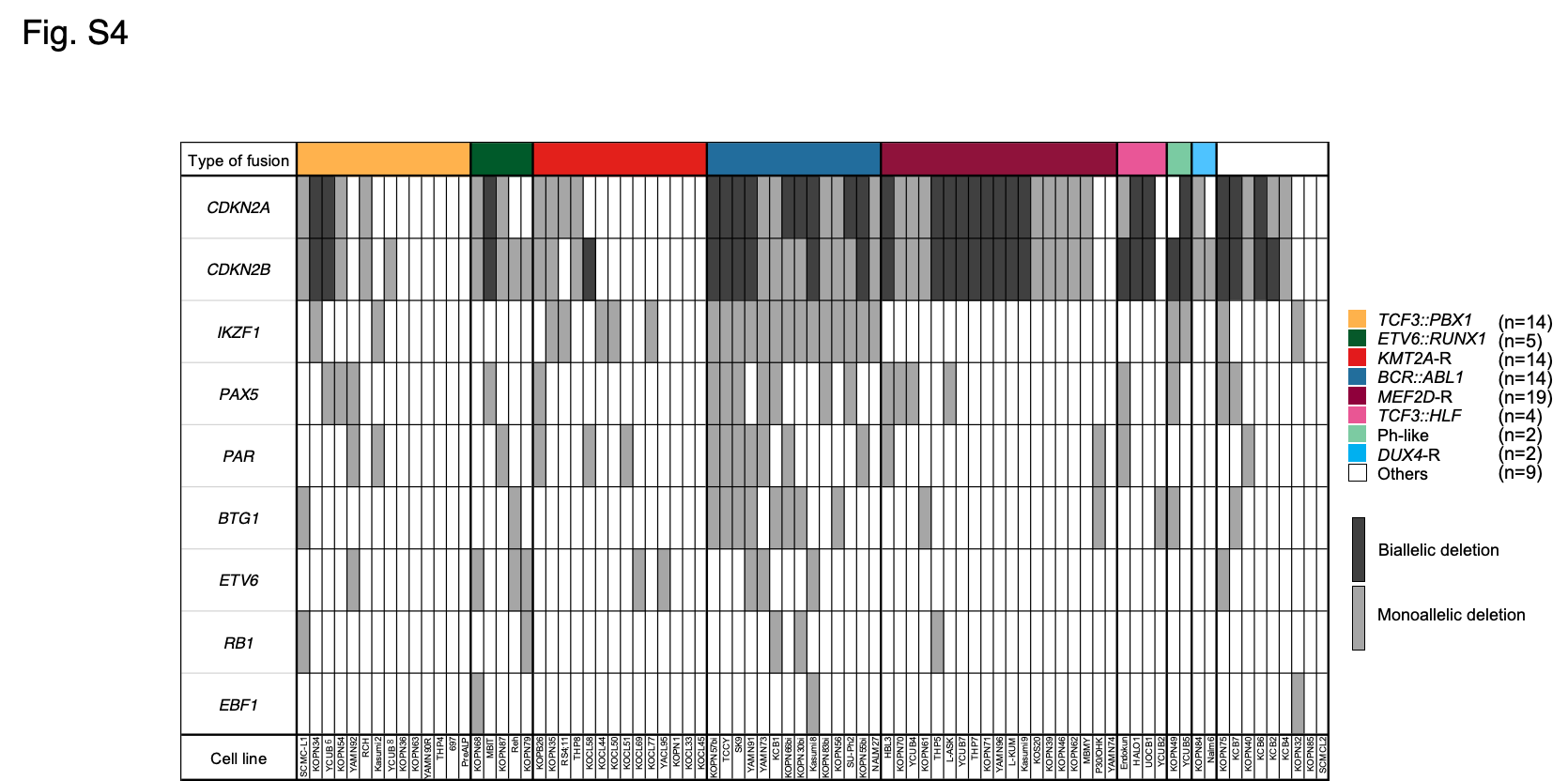

Supplement: Supplementary file 2 — Figure S1. [file CAM4-14-e70736-s002.docx]
